# Supplementary material for: Apparent Yield Stress of Sputum as a Relevant Biomarker in Cystic Fibrosis
Source: Cells. 2021 Nov 10;10(11):3107. doi: 10.3390/cells10113107 (PMC8619720; doi:10.3390/cells10113107)
Supplement: Supplementary file 1 [file cells-10-03107-s001.zip › cells-1440754-supplementary.pdf]

cells

MDPI

Supplementary material

Apparent yield stress of sputum as a relevant biomarker in Cystic Fibrosis

Rosy Ghanem[1], Philippe Roquefort[2], Sophie Ramel[3], Véronique Laurent[1], Tanguy Haute[1], Tony Le Gall[1], Thierry Aubry[2*], Tristan Montier[1,4*]

Table of contents

Supplementary Figures..**Error! Bookmark not defined.**2

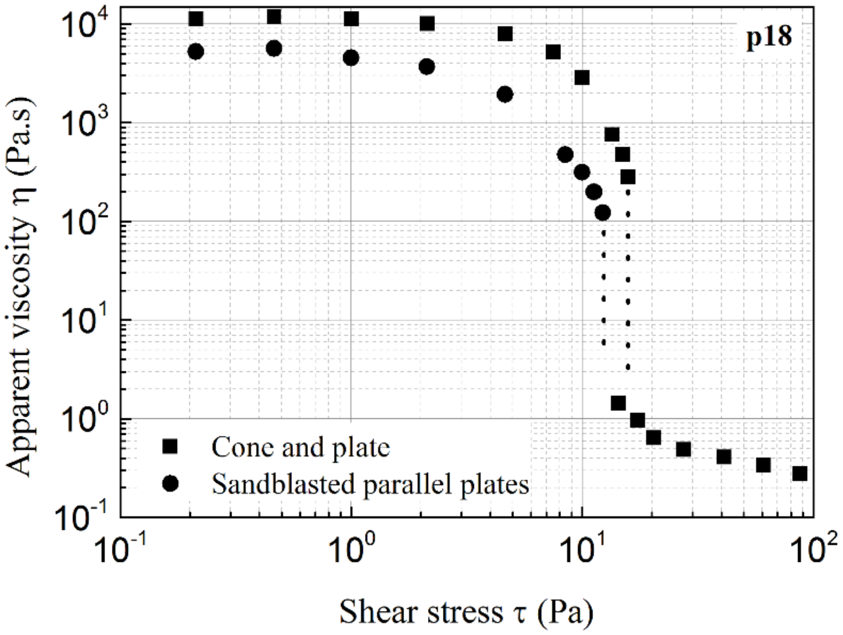

Figure S1: Investigation of possible slip effects: flow curve of p18 sputum using two different geometries; cone-plate (diameter: 50 mm, cone angle: 1°) and sandblasted parallel plates (diameter: 25 mm, gap: 1 mm).

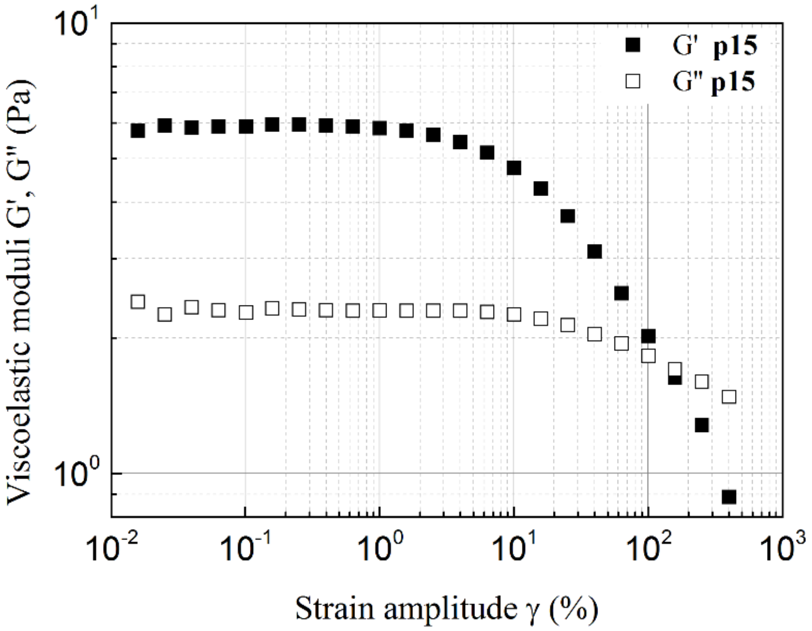

Figure S2: Determination of the linear viscoelastic domain: storage modulus G' and loss modulus G" as a function of strain amplitude (p15 sputum).
